# Supplementary material for: Factors Associated with the Work Engagement of Employees Working from Home during the COVID-19 Pandemic in Japan
Source: Int J Environ Res Public Health. 2021 Oct 6;18(19):10495. doi: 10.3390/ijerph181910495 (PMC8507692; doi:10.3390/ijerph181910495)
Supplement: Supplementary file 1 [file ijerph-18-10495-s001.zip › ijerph-1394379-supplementary.pdf]

International Journal of
Environmental Research and Public Health

MDPI

Supplementary Table S1. Prevalence ratio and 95% confidence interval values for high work engagement by whether or not employees were working from home using the Poisson regression model (univariable and multivariable analysis)

		univariable				multivariable			
		Working from home (+)		Working from home (-)		Working from home (+)		Working from home (-)	
		PR	95%CI	RR	95%CI	PR	95%CI	RR	95%CI
Age (per 10 years)		1.04	1.00-1.09	1.04	1.00-1.09	1.04	1.00-1.09	1.04	1.00-1.09
Gender (male/female)		1.04	0.94-1.15	1.03	0.98-1.09	1.07	0.95-1.20	1.06	1.02-1.13
Marital status	Married	Reference		Reference		Reference		Reference	
	Never married	0.80	0.72-0.9	0.77	0.72-0.82	0.85	0.75-0.97	0.85	0.79-0.91
	Widowed	1.39	0.95-2.02	1.28	1.08-1.53	1.14	0.77-1.68	1.19	1.00-1.43
	Separated	1.00	0.81-1.24	0.97	0.87-1.07	0.97	0.84-1.17	1.03	0.93-1.14
Shoulder pain (Yes/No)		0.98	0.89-1.08	1.18	1.12-1.25	1.06	0.95-1.13	1.04	0.97-1.11
Back pain (Yes/No)		1.16	1.05-1.28	1.17	1.10-1.24	1.06	0.94-1.17	1.03	0.96-1.11
Mental disorder (Yes/No)		1.43	1.29-1.58	1.42	1.34-1.50	1.33	1.18-1.50	1.34	1.25-1.44
Health behavior									
Physical activity	Increased	Reference		Reference		Reference		Reference	
	Unchanged	0.81	0.71-0.83	0.91	0.83-1.00	0.86	0.75-1.00	0.94	0.85-1.04
	Decreased	0.81	0.71-0.84	0.98	0.88-1.01	0.87	0.75-1.00	0.99	0.89-1.11
Sleeping hours	Increased	Reference		Reference		Reference		Reference	
	Unchanged	0.81	0.72-0.91	0.96	0.89-1.06	0.81	0.72-0.92	0.98	0.88-1.09
	Decreased	0.67	0.55-0.86	0.85	0.74-0.97	0.72	0.59-0.88	0.90	0.99-1.03
Not eating breakfast	Increased	Reference		Reference		Reference		Reference	
	Unchanged	0.87	0.73-0.91	0.94	0.82-1.09	0.67	0.50-0.90	0.87	0.75-1.01
	Decreased	1.03	0.83-1.28	1.01	0.81-1.20	0.90	0.63-1.26	0.91	0.76-1.09
Drinking alcohol	Increased	Reference		Reference		Reference		Reference	
	Unchanged	0.98	0.80-1.19	0.98	0.89-1.08	1.06	0.92-1.24	0.96	0.86-1.06
	Decreased	1.09	0.86-1.39	0.98	0.88-1.10	1.07	0.91-1.26	0.97	0.87-1.01
Smoking	Increased	Reference		Reference		Reference		Reference	
	Unchanged	0.98	0.82-1.18	1.01	0.87-1.17	1.02	0.82-1.26	0.97	0.83-1.14
	Decreased	0.89	0.72-1.11	1.30	0.95-1.34	1.02	0.79-1.32	1.04	0.87-1.25
Interacting									
With supervisors	Worse	Reference		Reference		Reference		Reference	
	Unchanged	1.49	1.23-1.81	1.50	1.33-1.69	1.24	0.98-1.57	1.37	1.18-1.59
	Better	2.12	1.65-2.70	2.16	1.82-2.52	1.61	1.17-2.22	1.76	1.42-2.18
With co-workers	Worse	Reference		Reference		Reference		Reference	
	Unchanged	1.49	1.21-1.89	1.33	1.17-1.50	1.20	0.93-1.59	1.00	0.85-1.17
	Better	1.40	1.58-2.68	1.86	1.59-2.20	1.31	0.93-1.83	1.25	1.00-1.55
Working hours	<40h/week	Reference		Reference		Reference		Reference	
	≧40h/week	0.75	0.67-0.87	0.79	0.95-0.84	0.84	0.74-0.95	0.83	0.77-0.88
Socioeconomic status									
Education (≦12y/>12y)		1.19	1.08-1.31	1.06	1.0-1.13	0.97	0.84-1.13	1.07	1.01-1.14
Household income (million JPY/year)	-2.99	Reference		Reference		Reference		Reference	
	3.00-7.99	0.85	0.71-1.02	1.01	0.93-1.09	0.94	0.77-1.15	1.01	0.93-1.10
	8.00-	0.99	0.82-1.19	1.11	1.01-1.21	1.05	0.85-1.31	1.08	0.97-1.19
	Unknown	0.89	0.71-1.11	0.98	0.89-1.08	0.94	0.74-1.19	0.95	0.86-1.05
Employee status	Permanent	Reference		Reference		Reference		Reference	
	Precarious	1.06	0.93-1.22	1.02	0.96-1.08	0.98	0.84-1.15	0.94	0.87-1.02
	Other	1.47	1.26-1.71	1.39	1.29-1.51	1.27	1.07-1.52	1.25	1.15-1.37

CI, confidence interval; h, hour; JPY, Japanese Yen; PR, prevalence ratio; y, year.
Note: The results are adjusted for age, gender, marital status, family number per household, shoulder/back pain, mental disorder, health behaviors (physical activity, sleeping hours, not eating breakfast, drinking alcohol, and smoking), interactions (with supervisors and co-workers), working hours, type of job, location, and socioeconomic status (education, household income, and employee status).
